# The Emerging Clinical Relevance of Artificial Intelligence, Data Science, and Wearable Devices in Headache: A Narrative Review

**DOI:** 10.3390/life15060909

**Published:** 2025-06-04

**Authors:** Antonios Danelakis, Anker Stubberud, Erling Tronvik, Manjit Matharu

**Affiliations:** 1 Department of Computer Science, NTNU Norwegian University of Science and Technology, 7030 Trondheim, Norway; 2 NorHead Norwegian Centre for Headache Research, 7030 Trondheim, Norway; anker.stubberud@ntnu.no (A.S.); erling.tronvik@ntnu.no (E.T.); manjit.matharu@nhs.net (M.M.); 3 Department of Neuromedicine and Movement Sciences, NTNU Norwegian University of Science and Technology, 7030 Trondheim, Norway; 4 St. Olavs University Hospital, 7030 Trondheim, Norway; 5 Headache and Facial Pain Group, UCL Queen Square Institute of Neurology and National Hospital for Neurology and Neurosurgery, London WC1N 3BG, UK

**Keywords:** artificial intelligence, machine learning, deep learning, generative AI, data science, wearable devices, headache research, ICHD criteria

## Abstract

This narrative review introduces key concepts in artificial intelligence (AI), data science, and wearable devices aimed at headache clinicians and researchers. PubMed and IEEEXplore were searched to identify relevant studies, and these were reviewed systematically. We identified six primary research topics. First, the most common application of AI and data science is in the diagnosis of headache disorders, with reported accuracies of up to 90%. Second, AI and data science are used for predicting headache disease trajectories and forecasting attacks. Third, prediction of treatment effects and data-driven individualization of treatment prescription demonstrate promising results, with accuracies ranging from 40% to 83%. Fourth, AI and data science can uncover hidden information within headache datasets, offering clinicians deeper insights. Fifth, wearables, combined with AI and data science, can improve remote monitoring and migraine management. Lastly, user experience studies indicate strong interest from both clinicians and patients in adopting these technologies. The potential applications of AI, data science, and wearable device technologies in headache research are vast. However, many studies are small pilot studies, and models often suffer from poor performance, limited reporting, and lack of external validation, which impede generalizability and clinical implementation.

## 1. Introduction

This review is intended to serve as an introductory guide for clinicians and researchers working in the headache field, offering an overview of artificial intelligence (AI), data science, and wearable devices and their applications in headache. We begin by providing a foundational introduction to AI, data science, and wearable devices, outlining essential concepts, definitions, and interconnections. Next, we present a comprehensive literature review on the use of AI, data science, and wearables in headache research, highlighting key clinical and technical aspects. Finally, we discuss challenges and opportunities for future research.

### 1.1. Artificial Intelligence

**Figure 1:**
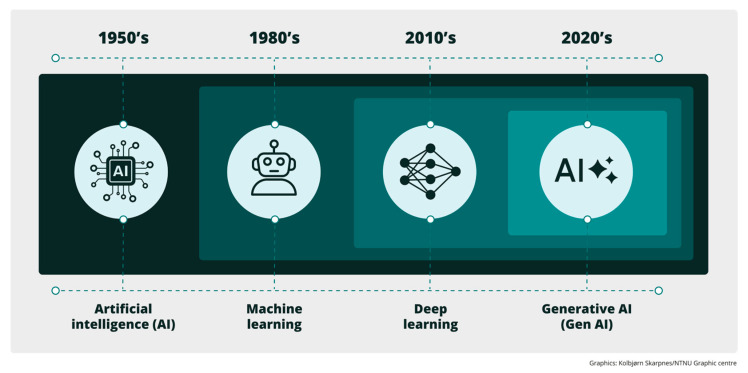
AI components overview.

AI is a field of computer science dedicated to developing systems or machines capable of performing tasks that typically require human intelligence [1]. AI encompasses three major components: (1) machine learning (ML), (2) deep learning (DL), and (3) generative AI (GenAI), as shown in Figure 1. 

ML is the core of AI and utilizes computational models that learn and improve by “learning” data [2]. Two primary types of ML are commonly employed in medical research: (i) supervised learning, in which algorithms are trained on data annotated (labeled) with the ground truth to make predictions or decisions; (ii) unsupervised learning, which works with non-annotated (unlabeled) data to uncover hidden patterns or structures within datasets [2]. However, there are two more secondary ML types that can rarely be found in headache research: (i) reinforcement learning, where a model is trained through interactions with an environment, receiving rewards or penalties depending on its actions; (ii) self-supervised learning, which enables models to train on unlabeled data by generating labels from the inherent information within the data itself.

DL is a subset of ML that uses multiple interconnected layers of artificial neurons (hence the term “deep”) to automatically learn patterns and features from data [3]. This is inspired by the structure and function of the human brain. The learning process progresses by adjusting the strengths of connections between neurons. Finally, generative AI comprises a subset of DL models that can learn underlying patterns and structures of data and use this knowledge to generate new and plausible data [4]. Notably, the emerging large language models (LLMs) [5], such as those used by latest versions of ChatGPT (GPT-4.1 mini) and Gemini (Gemini 2.5 Pro), fall within this category. For a comprehensive glossary of key AI concepts and terminology, the reader is referred to [6].

Previous research has suggested that AI could provide numerous advantages in headache research, including enhanced diagnostic accuracy, personalized treatment, identification of previously unknown precipitating factors of migraine attacks, and early prediction of future disease status and treatment efficacy capabilities [6]. Moreover, AI can identify correlations between clinical variables and specific phenotypes, as well as exploring interactive effects. This could provide a deeper understanding of headache disorders and contribute to cost reduction, minimization of medical errors, and an increase in patient safety [7]. Consequently, AI holds significant potential to improve both patient care and clinical outcomes for individuals suffering from headache disorders. As AI continues to advance, they are expected to play an increasingly critical role in transforming both research and clinical practices into the headache domain. However, a key challenge remains in fostering acceptance [8] of AI in the medical community, as it is often perceived as a “black box” tool, hindering its integration into clinical practice.

To evaluate AI models, it is important to validate the performance of the trained models on “unseen” data. This is typically carried out by splitting the data into a training set (data used for training the models), a validation test (used for temporary validation and optimization), and a testing set (unseen data that is used to evaluate the performance of the model). The test set is particularly important to ensure that the model is capable of transferring what it has learned to new and unseen data. A series of metrics can be used to evaluate AI models. We refer to a recent review for a detailed overview of these [6]. Additional metrics also discussed in the publications of this review are summarized in Table S1 of Supplementary Materials.

### 1.2. Data Science

Data science is increasingly valuable in headache research. It combines principles from statistics, computer science, and domain expertise to extract meaningful insights and knowledge from structured or unstructured data. The data can include, but is not limited to, clinical records, registry data, digitized self-reported questionnaires and scales, medical images, medication history, patient journals, and data captured or provided by external (non-wearable) devices like personal computers, smartphones, or tablets [9].

Data science encompasses a series of key processes such as data collection and acquisition, data cleaning and preprocessing, data visualization, and data analysis [9]. The analysis utilizes methodologies to identify hidden patterns, trends, and relationships in the collected and preprocessed data. The outcomes of the analysis can help clinicians better understand the underlying mechanisms of headache disorders. This, in turn, allows the generation of specific data-driven hypotheses. Additionally, data science facilitates the testing of these hypotheses and the extraction of meaningful inferences. By processing these data using advanced statistical and mining procedures, disorder diagnosis and monitoring can be conducted while treatment management can be refined [10]. This can be achieved either independently or in combination with more advanced AI-driven approaches.

### 1.3. Wearable Devices

A wearable device is a portable, easy-to-use electronic gadget worn on the body that monitors, tracks, captures, or manages various physiological and health metrics [11]. Assisted by integrated sensors, these devices continuously gather real-time data, such as heart rate, blood pressure, physical activity, sleep patterns, or even blood biomarkers. Most devices are accompanied by a mobile phone application that facilitates the interaction between the device and the user. In addition, wearables support remote monitoring, which enhances patient engagement and provides researchers with valuable longitudinal data that may facilitate an understanding of disease mechanisms and treatments [12].

**Figure 2:**
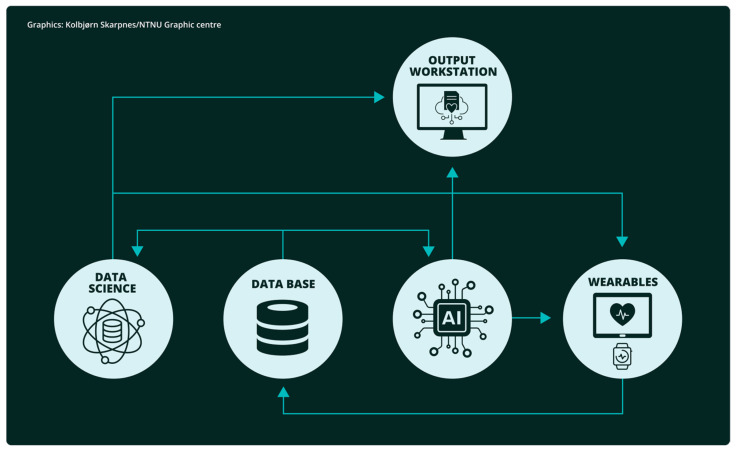
Relation and interaction among AI, data science, and wearable devices.

AI, data science, and wearable devices are not independent entities when it comes to medical applications; they typically, at least partly, coexist and interact with each other. For example, wearable devices collect data, which is then processed using data science techniques to extract initial insights. Finally, AI may be employed to train models with this data and carry out more complex operations. Such a pipeline is illustrated in Figure 2.

## 2. Methods

This is a narrative review that incorporates a systematic literature search to support a comprehensive and critical appraisal of the emerging clinical relevance of AI, data science, and wearable devices in headache. We searched PubMed and IEEEXplore for relevant literature using a combination of keywords to identify articles evaluating artificial intelligence, data science, and wearable devices in primary and secondary headache disorders. In the section reviewing AI, we build on a recent systematic literature review of AI in headache [6] and updated this with a literature search from April 2024 to January 2025. The following search terms were used on both databases: *(headache) OR (migraine) OR (tension-type headache) OR (trigeminal autonomic cephalalgia) AND (machine learning OR artificial intelligence). For data science and wearable devices, we searched PubMed and IEEEXplore from their inception to January 2025 using the following terms: ((headache) OR (migraine) OR (tension-type headache) OR (trigeminal autonomic cephalalgia)) AND (“data science”); and ((headache) OR (migraine) OR (tension-type headache) OR (trigeminal autonomic cephalalgia)) AND (wearable OR mHealth)*.

Publications were considered eligible for inclusion in this review if they were original publications in the English language that used any AI methodology, data science approaches, and/or wearable devices applied to any type of primary or secondary headache disorders as defined by the International Headache Society criteria [13,14].

In total, 879 records were identified, whereof 88 AI-related records in PubMed and 45 in IEEEXplore, 135 data science-related records in PubMed and 231 in IEEEXplore, and 325 wearable devices-related records in PubMed and 55 in IEEEXplore. Of these, 46 were removed by automated duplicate identification using EndNote 21, and an additional 3 duplicates were identified manually.

**Figure 3:**
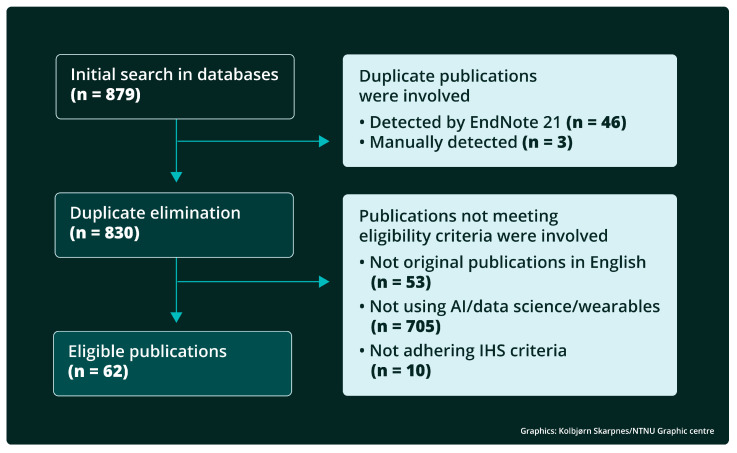
Filtering out the pipeline of the database search for this review. The term IHS is used for “International Headache Society”. The term REN is used for “remote electrical neuromodulation”.

The title and abstract of the remaining 830 records were screened using the above eligibility criteria, and 243 records were found to be potentially eligible based on their title and abstract and were reviewed in detail. Among these 243 records, 62 publications met the eligibility criteria and were included in this review. The above selection process is described in more detail in Figure 3.

For each area of interest (AI, data science, or wearable devices), the identified publications were categorized into thematic topics with respect to their clinical application. The following clinical applications were identified for AI: 1. diagnosis, 2. treatment, 3. prediction of future disease status and forecasting, 4. analysis, and 5. user experience. Clinical applications for data science were: 1. diagnosis, 2. monitoring, 3. treatment, 4. analysis, and 5. user experience. Finally, for wearable devices, the main clinical applications were: 1. monitoring, 2. analysis, 3. prediction of future disease status and forecasting, 4. Treatment, and 5. user experience.

Within each of the themes and clinical applications, the following core technical approaches were identified for AI: 1. ML, 2. DL, 3. Statistical analysis, and 4. LLMs. Similarly, for data science, it was: 1. statistical analysis, 2. fuzzy logic, 3. ML, 4. data capturing and visualization, and 5. DL. Finally, for wearable devices: 1. statistical analysis, 2. data capturing, 3. ML, and 4. DL.

## 3. Results

### 3.1. Artificial Intelligence

**Diagnosis:** Diagnostics, classification, and phenotyping of headaches appear to be the most common application of AI, and 26 such studies were identified in a recent review [6]. Among these 27 studies, the labeling of headache disorders was typically based on the ICHD-3 classification, and a variety of input features were used, including both clinical and phenotypic data, as well as paraclinical data, to train predictive models. In our updated literature search, the same patterns were observed. Input data for the models included electroencephalography (EEG) [15,16,17,18], medical imaging [19,20,21,22,23,24], headache-related clinical and medical data in tabular format [25,26,27,28,29,30,31,32], patient self-reported data [33,34,35,36], genetic data [37], and behavioral data [16]. Most studies focused on diagnosing migraine, while others have looked at other primary headaches like tension-type headaches [22,25,30,33,34,35,36], trigeminal autonomic cephalalgias [25,33], or even secondary headaches [33]. The number of participants in these studies varied significantly, ranging from just two individuals [21] to more than 43,000 [37]. Classical ML models were the predominant technical approach used, while one study used an unsupervised approach [36], and four used DL [19,24,28,35]. Diagnostic accuracy across these studies ranged from 62% [37] to 99.7% [17]. The most recent study, though, showcased that using ML to diagnose migraine based on genetic data, instead of the currently established polygenic risk scoring approach, achieves statistically significantly better results [37]. Although the diagnostic performance of many of these studies is high, a recognized, inherent limitation of classifying criteria-defined diseases, such as migraine, is that the accuracy cannot surpass that of the classification system [38].

**Treatment:** In the 2024 review, six studies using AI for the prediction of treatment effects were identified [6]. Among these, one study predicted response to calcitonin gene-related peptide (CGRP) treatments for migraine (accuracy from 70% to 84%) [39], one predicted the effect of non-steroidal anti-inflammatory drugs (NSAIDs) as acute treatment with an accuracy of 74% [40], and one demonstrated prediction of the preventive effect of verapamil for cluster headache (AUC: area under receiver operating characteristic curve score equals to 0.689) [41]. In addition, one study validated the concept of using prescriptive inference models to make predictive treatment recommendations and rankings for chronic migraine [42]. Since the 2024 review, four papers investigating the prediction of treatment response have been published.

Two studies utilized clinical records and self-reported data from 173 and 4260 patients, respectively, to predict the effect of migraine preventives. In the first study, a multi-dimensional Bayesian classifier was used to predict the effect of botulinum toxin type A (BoNT-A) [43]. It reported average treatment response prediction accuracies of 73%, 83%, and 77% across the three treatment stages; however, there were no details of test set evaluation. The second study utilized the TabNet DL architecture to convert the data to different representations, which were then used for training ML predictors using the H2OAutoML pipeline [44,45]. Fifteen percent of the data was kept out as a test set, while the remaining 85% was validated using 5-fold cross-validation. The study achieved prediction accuracies of 80%, 73%, 66%, 63%, 63%, 60%, and 60% for CGRP mAb, beta-blockers, gabapentin, topiramate, onabotulinumtoxinA, tricyclic antidepressants and verapamil, respectively.

Two additional studies aimed to predict the efficacy of migraine treatments [46,47] using imaging data. In one of these, the focus was on CGRP mAb treatment [46]. A total of 336 patients were used to train and validate various machine learning models, with an external cohort of 93 patients used for testing. The random forest model achieved the highest prediction accuracy, ranging from 40% to 65% in the testing cohort, depending on the month of prediction after treatment. In contrast, the other study focused on identifying potential neuroimaging biomarkers to predict responses to non-steroidal anti-inflammatory drugs for episodic migraine [47]. To this end, resting-state fMRI images from 118 participants were used, along with a separate testing cohort of 39 participants. Multiple machine learning models were evaluated, with the random forest model once again yielding the best performance, achieving a prediction accuracy of 61% on the held-out test cohort.

**Prediction of future disease status and forecasting:** In the 2024 review, a handful of studies using AI for the prediction of future disease status and headache forecasting were identified. Noteworthy among these was an ML model that, with an accuracy of 87%, could predict which patients with medication overuse headache would become medication overuse-free using demographic and clinical data collected through questionnaires [48]. In addition, in a recently published study, an ML model was created to predict the new onset of migraine using a combination of genetic and clinical/demographic data [49]. Importantly, the models combining genotype data and clinical data outperformed those using only genotype data or only clinical data, suggesting that genotypic-phenotypic interactions may partake in the new onset of migraine.

**Analysis:** AI has also been used to improve our understanding of headache disorders. For example, in a 2024 study, the Boruta feature importance algorithm was used to detect possible associations between genetic and self-reported headache data with migraine [50]. A total of 459 individuals participated in the study, which concluded that, although some genetic variants are possibly associated with migraine, the self-reported headache data showed higher associations. Another 2024 study also used a combination of self-reported and clinical data of 61,826 individuals to identify factors associated with seeking medical care for migraine [51]. Random forest and logistic regression architectures were used and demonstrated that migraine sufferers were more likely to seek medical care with increasing interictal burden, disability, and allodynia. Finally, one study used a multivariate logistic regression model to analyze factors influencing mild depression in 178 individuals with migraine without aura [52].

**User experience:** A 2024 study examined doctors’ experience with LLMs in the context of answering common migraine-related questions [8]. Five different LLMs were tested with 30 queries each. The responses were reviewed by three independent migraine specialists, who rated them on a simple scale: 0 for inappropriate and 1 for appropriate. The results showed that most of the responses were accepted positively and that the expert physicians considered LLMs to be valuable tools for assisting in clinical practice in the field of headache.

### 3.2. Data Science

**Diagnosis:** Several data science publications study data-driven diagnostics [53,54,55], much similar to the AI approach. However, the data science approach typically uses decision rules such as IF-THEN-ELSE. All these works are focused on migraine. They exploited headache-related clinical data [53,55] or medical images like PET [55] on cohorts with the number of participants ranging from 61 [54] to 705 [55]. Two studies use a fuzzy logic algorithm for creating data-driven decision rules [53,55], while one study [54] examined a comprehensive big data analytics protocol (CBDA) in combination with ML predictors. The CBDA protocol was used for converting the initial raw data into a different representation, which is then used to train ML diagnostic models. The techniques achieve prediction accuracies of over 90%.

**Monitoring:** Two studies were identified that used data science methodologies to collect and monitor headache data through web-based interfaces [56,57]. Another study used a mobile application for the same purpose [58], while a fourth study utilized a hybrid approach, which involves a mobile application to be used by the patients for data capturing and communication with a web-based platform to be used by the clinicians for data analysis [59]. In all cases, the captured data is self-reported headache diary information, and migraine was the most targeted disorder [57,58], but other primary or secondary headaches were also considered [56,59]. The studies utilized data visualization techniques to help clinicians monitor and keep track of the trends of the patients’ disorders. In addition to the data visualization, three studies also implemented data-driven rule-based algorithms to generate appropriate alerts on the patient’s symptoms [57,59] or classify the headache type [58]. According to the aforementioned studies, the implementation of an electronic monitoring tool improved the outcome of patients suffering from headaches.

**Treatment:** A 2021 publication conducted a pair-wise comparison statistical analysis using data from the DrugBank [60] dataset to examine the interactions among different acute headache medications [61]. According to the authors, the proposed approach could save clinicians a lot of time since reviewing and reconciling patients’ headache medications is cumbersome.

**Analysis:** In a 2023 study, the resting-state fMRI image data of 100 individuals were used for studying the associations between episodic migraine and cortical and subcortical alterations [62]. A linear regression predictor of migraine frequency and duration was developed. Although the performance of the model for predicting frequency was significant (ICC: intra-class correlation equals to 0.33 with SD: standard deviation equal to 0.13, and MAE: mean absolute error equals to 2.97 with SD equal to 0.57) this was not the case for the duration. The model was assessed on a validation set without being clarified whether this was a held-out set. In a 2013 study, density estimation algorithms were used to analyze data to associate migraine with other comorbidities [63]. Ambulatory and hospitalization records of 116,136 patients were used as an input to the algorithms. The algorithms found migraine to be related to various mental, cardiovascular, neurological, gastroenterological, hepatological, musculoskeletal, metabolism, endocrinology, and otolaryngological disorders. Another study used brain MRI images of 51 subjects and a decision tree regression model on the extracted image features to explore associations between abnormalities in the brain structure and new daily persistent headache (NDPH) [64]. The research suggests that damage to the superficial white matter structure constitutes one of the pathological features of NDPH.

**User experience:** For data science to make a meaningful impact in headache research, both patients and clinicians must have confidence in using external digital tools for data collection and analysis [65]. To this end, there are publications that study how these tools prioritize a positive user experience by focusing on both feasibility and usability, ensuring accurate data registration and fostering engagement from both patients and clinicians [66,67,68,69,70,71]. One study investigated what individuals with migraine considered to be important features of migraine tracking [66]. The study used the grounded theory approach to analyze free-text inputs in a progressive muscle relaxation mHealth app among 288 patients. It was found that most patients wish to expand on information beyond what can be inputted into specific icons in the app, that they want to monitor non-headache-related symptoms, that they want to track non-pharmacological treatment, and they want to track possible triggers and relievers of their migraine. Three other publications have evaluated the feasibility, usability, user acceptance, user engagement, and user satisfaction of different mobile migraine management applications [67,68,69]. Each study explored a different application that captured self-reported headache-related data. According to those works, patients were highly engaged with using data acquisition digital health solutions for headache management, and those can potentially relieve their burden better than typical approaches. An additional 2024 study supported the results of the three previously mentioned studies by adopting a similar approach to assessing 535 migraine patients’ satisfaction when using an electronic headache diary [70]. The electronic diary was well-liked and was considered to increase accessibility. Finally, another 2024 study utilized a random forest algorithm combined with logistic regression on migraineurs self-reported data to extract potential reasons that those patients hesitate to seek care for migraine [71]. Data from a total of 58,403 patients from the OVERCOME (US: United States) [72] was used. The study concluded that the social context of migraines, such as stigma, plays a key role in the reluctance to seek care.

### 3.3. Wearable Devices

**Monitoring:** A currently under-research application of wearables in headache management is the concept of continuous monitoring through a headache digital twin [73]. To this end, a system for real-time monitoring of migraine patients, including cloud computing and wearable devices, to collect a wide range of multimodal data and then process it using AI, ML, and DL to command downstream personalized actions has been proposed. The concept is still only theoretical, but it provides an idea of the potential of the digital twin approach in the headache field.

**Analysis:** Wearables are used to explore potential correlations between externally measured data and headaches. One study used an analysis of variance (ANOVA) model to identify specific key EEG-related predictors for migraine attacks [74]. The study concluded that a well-established visual attention event-related brain potential can be used as an important predictor for migraine. Two other studies collected physical activity or activity energy expenditure data, which was then associated with the occurrence of migraine, tension-type, and cluster headaches. One of the studies found that during the migraine and tension-type headache days, patients slept more, had reduced physical activity, and had lower maximum heart rates [75]. Moreover, individuals with migraine face a greater burden in everyday life than patients suffering from tension-type headaches. The last study observed decreased activity energy expenditure during the pre-ictal and cluster headache phase, although hyperactivity was initially hypothesized [76].

**Headache forecasting by wearable data:** A series of studies have evaluated the use of physiological data captured from wearables to predict migraine attacks. Inputs for the models were somatosensory evoked potentials (SEP) [77], EEG [78], the combination of accelerometer data, thermometer data, electrodermal activity data, blood volume pulse, heart rate and heart rate variability [79], self-reported headache diary data combined with muscle tension, peripheral skin temperature and heart rate [80] and the combination of pulse rate, physical activity data, skin temperature and electrodermal activity data [81]. The study cohorts vary from 7 [77] to 18 [78] patients. The acquired data were utilized by ML models [79,80,81] or DL models [77,78] to predict attacks. The techniques achieved prediction accuracies ranging from 62% [80] to 84% [79] on the respective testing sets.

**Treatment:** Wearables are used to deliver different types of behavioral treatments, such as biofeedback [82,83,84,85,86]. These studies are primarily pilot, usability, and feasibility trials, but two of the larger studies suggest that such treatments may be effective [82,83]. In the first study, participants with migraine were randomized to heart rate variability biofeedback or a waitlist control [82]. The treatment was considered feasible and acceptable, but no difference between groups in the primary clinical outcome of Migraine-specific quality of life was observed. In the second study, 84 individuals with migraine were randomized to an app-based surface electromyographic biofeedback (sEMG-BF) [87] intervention or usual care [83]. Those randomized to biofeedback showed improvements in disability and quality of life but no change in migraine frequency.

**User experience:** An important aspect of improving the user experience when it comes to utilizing wearable devices in headache research is enhancing feasibility and usability, meaning that those devices should be user-friendly and convenient for the patients and the clinicians to use [65]. Four of the previously mentioned biofeedback studies have investigated this [82,84,85,86]. In two of those studies, the usability and feasibility of biofeedback approaches for pediatric migraines were investigated [84,85]. Both approaches utilize a smartphone application and wearable sensors. Both studies demonstrated high usability. However, patients in the second study reported difficulties with burden, remembering to wear the sensor, and requiring migraine attack prediction information to be displayed. The final study assessed the feasibility and usability of a biofeedback approach against migraine over a period of four weeks in an adult cohort this time, with the users reporting an overall positive experience, good usability, and a high adherence rate [86].

### 3.4. Statistical Summary

**Figure 4:**
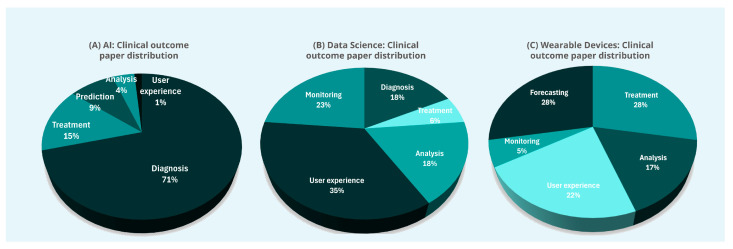
Distribution of new (**A**) AI, (**B**) data science, and (**C**) wearable devices publications included in this review based on their clinical outcome.

**Figure 5:**
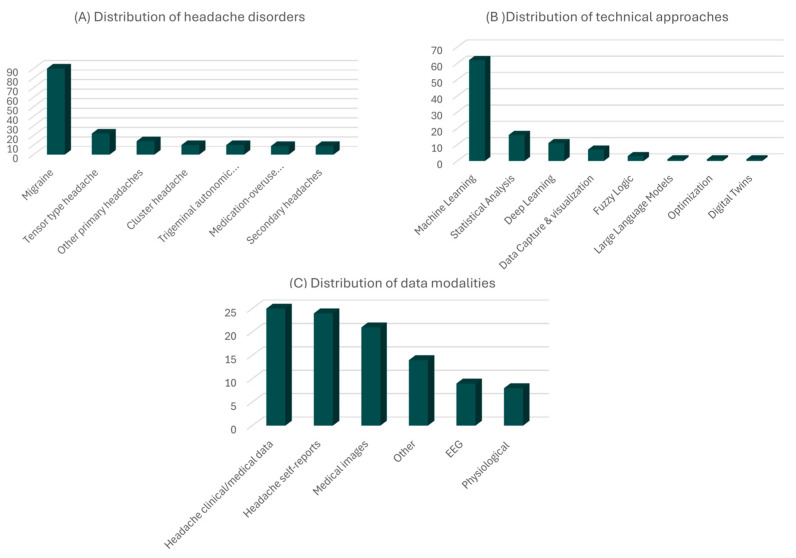
Distribution of the (**A**) headache disorders, (**B**) technical approaches, and (**C**) data modalities studied by the new publications reviewed in this study. Possibly more than one technical approach, data modality, and disorder were examined for each publication.

The key applications of AI, data science, and wearable devices publications are illustrated in Figure 4. Migraine, as the most common and economically burdensome primary headache disorder [88], continues to be the primary focus of research efforts, as shown in Figure 5A. The obvious preference for ML-based approaches is illustrated in Figure 5B.

The data utilized in these studies predominantly consists of easily accessible information, such as data collected through sensors (wearable or not), self-reported by patients, or retrieved from medical registries. In contrast, data types with inherent acquisition difficulties, such as genetic information, are less frequently used, as indicated in Figure 5C.

The numerical data of the related publications can be found in Tables S2 and S3 of Supplementary Materials. These tables also take into consideration the publications included in the 2024 review [6]. The overview and more information of the new publications, which are presented in the current literature review, can be found in Tables S4–S6 of Supplementary Materials.

## 4. Discussion

Artificial intelligence, data science, and wearable devices are increasingly being integrated into headache research. These advanced tools have the potential to facilitate clinical workflows, enhance patient care, improve clinical outcomes, and support various aspects of headache management. However, alongside the promises these innovations bring, several key challenges remain that must be addressed to fully realize their potential in headache research.

AI and data science hold immense promise to improve headache research by analyzing large datasets to uncover new insights into headache pathophysiology, risk factors, and treatment responses [6]. Machine learning models, when trained on large-scale, high-quality data, have demonstrated considerable diagnostic accuracy and enhancement of personalized treatment strategies [6,12]. In the clinical setting, AI can streamline physicians’ workflows by automating routine tasks, such as the categorization of patient-reported symptoms and the integration of complex data from multiple sources, giving them the opportunity to make more informed decisions. This could potentially lead to more timely, precise, and improved patient outcomes. In addition, the data capture capability introduced by wearable devices represents a potential benefit for headache research [12,89]. These devices allow for the real-time capture of headache-related and physiological data, offering dynamic visualizations and analysis, which enable the extraction of more insights into headache patterns over time.

This review uncovered an obvious preference for ML-based approaches. This can be attributed to the ability of ML models to detect complex interactive patterns within large datasets, a capability that surpasses the typically additive effects captured by traditional statistical approaches. Furthermore, ML models offer greater flexibility, ease of interpretation, and better match the scale of medical datasets currently in use. In contrast, DL models are often used as “black boxes” and require large volumes of data to yield reliable results, making them less practical in some clinical settings.

Despite the promising potential, several critical challenges hinder the widespread implementation and clinical integration of AI, data science, and wearables in headache research [1]. One of the primary concerns is the generalizability of the developed predictive models, largely due to the under-representation of clinically relevant subgroups. Many of the datasets used to train these models are of relatively small size, and important variables are often absent or inconsistently captured. Relatedly, external validation on independent, out-of-sample datasets is frequently inadequate. All the reasons outlined above introduce risks of selection bias and model overfitting, making these models unreliable in real-world clinical settings. Consequently, while the models may perform well on specific datasets, their predictive capabilities may not be effective across diverse populations. It needs to be emphasized here that the risk of biased algorithms and overfitted models is particularly significant in medical contexts, where diverse factors such as ethnicity and gender must be considered to ensure equitable and effective care for all patients. This limitation underscores the need for a cautious and nuanced interpretation of current findings. While current models show promise, their predictive accuracy remains suboptimal, leaving a good margin for further research and improvements. Enhancing the accuracy of these models could be pivotal in personalizing treatments and improving patient treatment efficacy.

To overcome these challenges, a focused effort to collect larger and more diverse multimodal datasets is necessary. However, managing sensitive medical data presents significant challenges due to stringent legislation, such as the General Data Protection Regulation (GDPR) [90], the European Union Medical Device Regulation, and the US Federal Food, Drug, and Cosmetics Act and its 21st Century Cures Act [91,92,93]. One promising approach to address this challenge is the use of generative AI technologies to create realistic synthetic data. This approach can augment existing datasets, enhancing the robustness and generalizability of predictive models while ensuring compliance with privacy regulations and ethical standards. However, the use of synthetic data must be approached with caution to ensure that these datasets accurately represent real-world variability and do not introduce artificial biases into the models.

Furthermore, while wearable devices offer exciting opportunities for continuous and remote monitoring, their integration into clinical pathways is not without challenges. Signal artifacts, variability in calibration, and inconsistent patient adherence can all compromise data quality, limiting their immediate utility [94]. Addressing these issues in future work will be essential to ensure that these technologies are both effective and equitable in real-world clinical care.

Although the results of this review suggest that the scientific and clinical communities are eager to embrace these advanced technologies, there is still a significant gap between human understanding and AI. Bridging this gap is also a challenge highlighted by this review. The ongoing development of Explainable AI (XAI) [95], which focuses on improving the transparency and interpretability of AI models, is expected to facilitate greater acceptance among healthcare professionals and patients alike. By providing clear insights into how AI-driven decisions are made, XAI can enhance trust and confidence in these novel technologies.

Finally, the design of future studies in headache research should combine innovative approaches that leverage AI, data science, and wearable technology with traditional clinical trial methodologies. The latter is crucial to guarantee that the developed models will be evaluated on out-of-sample datasets in order for their generalizability to be reliably assessed and to avoid the creation of overfitted models.

## 5. Conclusions

The use of AI, data science, and wearables in headache research is rapidly expanding, with applications spanning diagnostics, disease trajectory prediction, forecasting models, treatment effect analysis, data monitoring, user experience assessment, and the identification of underlying disorder patterns. There seems to be a strong desire for both patients and clinicians to incorporate such technologies in clinical practice, yet the clinical applicability and values of these technologies remain unclear at present. Many studies suffer from inadequate performance, incomplete reporting, and often lack external validation. Additionally, data availability and cumbersome legal protection and regulatory pathways can be obstacles. If these challenges are addressed, these technologies hold the potential to develop more effective models, clinical decision-support tools, and treatments, ultimately improving the management and understanding of headache disorders.

## Data Availability

No new data were created or analyzed in this study. Data sharing is not applicable to this article.

## Supplementary Materials

The following supporting information can be downloaded at: https://www.mdpi.com/article/10.3390/life15060909/s1. Table S1: Additional model evaluation metrics discussed in the new publications illustrated in this review. Table S2: Numerical data on the clinical outcome distributions of AI (including 2024 review studies [6]), data science and wearable device technologies. Table S3: Numerical data on the technical approaches, data modalities and headache disorders distributions of AI (including 2024 review studies [6]), data science and wearable device technologies. Table S4: New publications on AI included in the review. The table provides information regarding, where applicable, the headache disorder, the cohort, the data modality, the clinical outcome, the validation and testing dataset splitting, the technical methodology, the evaluation metric, the trial period and the performance reported by the corresponding publication. Table S5: New publications on data science included in the review. The table provides information regarding, where applicable, the headache disorder, the cohort, the data modality, the clinical outcome, the validation and testing dataset splitting, the technical methodology, the application used for gathering data, the trial period and the performance reported by the corresponding publication. Table S6: New publications on wearable devices included in the review. The table provides information regarding, where applicable, the headache disorder, the cohort, the data modality, the clinical outcome, the validation and testing dataset splitting, the technical methodology, wearable devices, the trial period and the performance reported by the corresponding publication.

## Abbreviations

The following abbreviations are used in this manuscript:

AI	artificial intelligence
ML	machine learning
DL	deep learning
GenAI	generative artificial intelligence
LLM	large language models
EEG	electroencephalography
CGRP	calcitonin gene-related peptide
NSAID	non-steroidal anti-inflammatory drug
BoNT-A	botulinum toxin type A
AUC	area under the receiver operating characteristic curve
CBDA	comprehensive big data analytics
ICC	intra-class correlation
SD	standard deviation
MAE	mean absolute error
NDPH	new daily persistent headache
US	United States
ANOVA	ANalysis Of VAriance
SEP	somatosensory evoked potentials
sEMG-BF	surface electromyographic biofeedback
GDPR	general data protection regulation
XAI	explainable artificial intelligence
