# Supplementary material for: The Emerging Clinical Relevance of Artificial Intelligence, Data Science, and Wearable Devices in Headache: A Narrative Review
Source: Life (Basel). 2025 Jun 4;15(6):909. doi: 10.3390/life15060909 (PMC12193817; doi:10.3390/life15060909)
Supplement: Supplementary file 1 [file life-15-00909-s001.zip › life-3644733-SupplementaryMaterials.pdf]

Supplementary Materials

Supplementary tables

Table S1. Additional model evaluation metrics discussed in the new publications illustrated in this review.

Metric	Area	Assessment	Definition
Youden's J Statistic	AI	Diagnosis/Prediction	A metric to evaluate model performance, calculated as (Sensitivity + Specificity – 1). It ranges from 0 to 1, where higher values indicate better performance.
Calibration Curve	AI	Diagnosis/Prediction	A plot that shows how well the predicted probabilities of a model align with the true probabilities.
Confusion Matrix	AI	Diagnosis/Prediction	A table which is used to evaluate the performance of a model, showing the number of true positives, true negatives, false positives, and false negatives.
NPV (Negative Predictive Value)	AI	Diagnosis/Prediction	The proportion of true negatives among all negative predictions made by the model.
FNR (False Negative Rate)	AI	Diagnosis/Prediction	The proportion of actual positives that are incorrectly characterized as negatives.
PPV (Positive Predictive Value)	AI	Diagnosis/Prediction	The proportion of true positives among all positive predictions made by the model.
Balanced Accuracy	AI	Diagnosis/Prediction	A metric to evaluate performance, particularly in imbalanced datasets. It is the average of Sensitivity and Specificity.
ICC (Intra-Class Correlation)	AI	Diagnosis/Prediction	A measure of the reliability or consistency of ratings or measurements made by different raters or tools.
MAE (Mean Absolute Error)	AI	Diagnosis/Prediction	An evaluation metric that calculates the average of the absolute errors between predicted and actual values, showing the magnitude of errors in the model.
MCC (Matthews Correlation Coefficient)	AI	Variable correlation	A metric which is used to assess the quality of models. It considers all four confusion matrix categories and ranges from -1 (perfect negative) to +1 (perfect positive), with 0 indicating random performance.
Mean Decrease Accuracy	AI	Variable importance	A metric used to assess the importance of a variable in machine learning models, by measuring the

 https://doi.org/10.3390/xxxxx

			decrease in model accuracy when a variable is permuted or removed.
Gini Index	AI	Variable importance	In decision tree-based models, the Gini index is used to evaluate variable importance by measuring how much a given variable reduces the impurity at each tree split. Variables that significantly reduce impurity are considered more important because they help the model make better decisions and predictions.
Regression Tree Weights	AI	Variable importance	Prediction weights that are assigned to each node in a regression tree model which indicates the importance of the node in the prediction, and thus, the importance of the respective variable.
z-score	Data science	Variable correlation	A statistical measure that indicates how many standard deviations a data point has from the mean of the distribution.
p-values	Data science	Variable significance	A measure of the evidence against a null hypothesis in statistical tests. A low p-value indicates strong evidence against the null hypothesis.
OR (Odds Ratio)	Data science	Variable correlation	A measure of association between an exposure and an outcome. It compares the odds of an outcome occurring in an exposed group to the odds in a non-exposed group.
Spearman Rank	Data science	Variable correlation	A non-parametric test that assesses how well the relationship between two variables can be described using a monotonic function.
β coefficients	Data science	Variable correlation	The coefficients that represent the relationship between predictor variables and the outcome variable, quantifying the impact of each predictor.
95% CI (Confidence Interval)	Data science	Variable significance	A range of values, derived from a sample, that is likely to contain the population parameter with 95% confidence.
q-values	Data science	Variable significance	A p-value used to correct for multiple comparisons in statistical tests.
Bonferroni test	Data science	Variable correlation	A statistical method used to correct multiple comparisons in hypothesis testing by adjusting p-values to control the family-wise error rate.

Table S2. Numerical data on the clinical outcome distributions of AI (including 2024 review studies [6]), data science and wearable device technologies.

**Artificial Intelligence**	
**Clinical Outcome**	**Number of Studies**
Diagnosis	49
Treatment	10
Prediction	6
Analysis	3
User experience	1
**Data Science**	
**Clinical Outcome**	**Number of Studies**
Diagnosis	3
Monitoring	4
Treatment	1
Analysis	3
User experience	6
**Wearable Devices**	
**Clinical Outcome**	**Number of Studies**
Monitoring	1
Analysis	3
Forecasting	5
Treatment	5
User experience	4

Table S3. Numerical data on the technical approaches, data modalities and headache disorders distributions of AI (including 2024 review studies [6]), data science and wearable device technologies.

**Artificial Intelligence, Data Science and Wearable Devices**	
**Technical Approaches**	**Number of Studies**
Machine learning	63
Statistical analysis	16
Deep learning	11
Data capture & visualization	7
Fuzzy logic	3
Large language models	1
Optimization	1
Digital twins	1
**Data Modalities**	**Number of Studies**
Headache clinical/medical data	25
Headache self-reports	24
Medical Images	21
EEG data	9
Physiological data	8
Other types of data	15
**Disorders**	**Number of Studies**
Migraine	91
Tension type headache	22
Cluster headache	10
Trigeminal autonomic cephalalgia	10
Medication overuse headache	9
Other primary headaches	14
Secondary headaches	9

Table S4. New publications on AI included in the review. The table provides information regarding, where applicable, the headache disorder, the cohort, the data modality, the clinical outcome, the validation and testing dataset splitting, the technical methodology, the evaluation metric, the trial period and the performance reported by the corresponding publication.

Nr	Title	Characteristics
1	MwoA auxiliary diagnosis using 3D convolutional neural network	**Disorders:** [Migraine]<br>**Cohort:** [125 subjects]<br>**Data:** [rs-fMRI image data]<br>**Outcome:** [Migraine diagnosis]<br>**Validation:** [5-fold] [No train/test split info]<br>**Core technical approach:** [Deep learning]<br>**Performance:** [Accuracy: 98.4%, Sensitivity: 96.67%, Specificity: 100%]
2	Classification of Headache Disorder Using Random Forest Algorithm	**Disorders:** [Primary headache]<br>**Cohort:** [850 subjects]<br>**Data:** [Headache clinical and medical records]<br>**Outcome:** [Headache diagnosis]<br>**Validation:** [5-fold] [No train/test split info]<br>**Core technical approach:** [Machine learning]<br>**Performance:** [Accuracy: 99.56%]
3	Subgrouping Factors Influencing Migraine Intensity in Women: A Semi-automatic Methodology Based on Machine Learning and Information Geometry	**Disorders:** [Migraine]<br>**Cohort:** [67 subjects]<br>**Data:** [Headache clinical and medical records]<br>**Outcome:** [Migraine diagnosis]<br>**Validation:** [8-fold] [No train/test split]<br>**Core technical approach:** [Machine learning]
4	Classification of Migraine Disease using Supervised Machine Learning	**Disorders:** [Migraine]<br>**Cohort:** [400 subjects]<br>**Data:** [Headache clinical and medical records]<br>**Outcome:** [Migraine diagnosis]<br>**Validation:** [No validation set info] [70/30 train/test split]<br>**Core technical approach:** [Machine learning]<br>**Performance:** [Accuracy: 94.16%, Precision: 93.9%, Recall: 94.2%, F1-score: 94%, AUC: 97.8%]
5	Migraine Prediction Using Deep Learning Model	**Disorders:** [Migraine]<br>**Cohort:** [400 subjects]<br>**Data:** [Headache clinical and medical records]<br>**Outcome:** [Migraine diagnosis]<br>**Validation:** [DL validation] [No train/test split info]<br>**Core technical approach:** [Deep learning]<br>**Performance:** [Accuracy: 99%, Precision: 99%, Recall: 99%, F1-score: 99%]
6	Developing an artificial intelligence-based headache diagnostic model and its utility for non-specialists' diagnostic accuracy	**Disorders:** [Primary and secondary headache]<br>**Cohort: [**4,050 subjects]<br>**Data:** [Headache self-reported patient data]<br>**Outcome:** [Headache diagnosis]<br>**Validation:** [10-fold] [70/30 train/test split]<br>**Core technical approach:** [Machine learning]<br>**Performance:** [Accuracy: 76.25%, Sensitivity: 56.26%, Specificity: 92.16%, Precision: 61.24%, F1-score: 56.88%]
7	Automatic classification of migraine and tension-type	**Disorders:** [Migraine and tension type headache]<br>**Cohort:** [160 subjects]

	headaches using machine learning methods	**Data:** [Headache self-reported patient data]<br>**Outcome:** [Migraine/ tension type headache diagnosis]<br>**Validation:** [3-fold] [No train/test split info]<br>**Core technical approach:** [Machine learning]<br>**Performance:** [Accuracy: 98%, Sensitivity: 100%, Specificity: 96%]
8	Identification of genetic susceptibility for Chinese migraine with depression using machine learning	**Disorders:** [Migraine]<br>**Cohort:** [489 subjects]<br>**Data:** [Genetic data]<br>**Outcome:** [Migraine analysis]<br>**Core technical approach:** [Statistical analysis and machine learning]<br>**Evaluation:** [Gini index]
9	Machine learning identifies factors most associated with seeking medical care for migraine: Results of the OVERCOME (US) study	**Disorders:** [Migraine]<br>**Cohort:** [61,826 subjects]<br>**Data:** [Clinical and medical records]<br>**Outcome:** [Migraine analysis]<br>**Core technical approach:** [Machine learning]<br>**Evaluation:** [Gini index]
10	Electroencephalographic signatures of migraine in small prospective and large retrospective cohorts	**Disorders:** [Migraine]<br>**Cohort:** [62 subjects]<br>**Data:** [EEG signal data]<br>**Outcome:** [Migraine diagnosis]<br>**Validation:** [4-fold] [No train/test split]<br>**Core technical approach:** [Machine learning]<br>**Performance:** [Accuracy: 87.2%, Precision: 85.2%, Recall: 97.5%, Specificity: 71.4%, AUC: 82.9%]
11	Multidimensional Bayesian Classifier for Predicting the Multi-stage Patient's Response to the BoNT-A Treatment for Migraine	**Disorders:** [Migraine]<br>**Cohort:** [173 subjects]<br>**Data:** [Headache clinical and medical records]<br>**Outcome:** [Migraine treatment response prediction]<br>**Validation:** [10-fold] [No test split used]<br>**Core technical approach:** [Machine learning]<br>**Performance:** [*1st treatment stage*: Accuracy: 79.45% ± 0.83%, Sensitivity: 81.22% ± 2.17%, Specificity: 75.10% ± 1.54%. *2nd treatment stage*: Accuracy: 82.57% ± 1.38%, Sensitivity: 77.63% ± 2.17%, Specificity: 83.21% ± 2.23%. 3rd *treatment stage*: Accuracy: 77.35% ± 0.45%, Sensitivity: 75.12% ± 1.05%, Specificity: 79.12% ± 1.25%]
12	Disrupted gray matter connectome in vestibular migraine: a combined machine learning and individual-level morphological brain network analysis	**Disorders:** [Migraine]<br>**Cohort:** [112 subjects]<br>**Data:** [MRI image data]<br>**Outcome:** [Migraine diagnosis]<br>**Validation:** [Nested CV: 5-fold] [No train/test split]<br>**Core technical approach:** [Machine learning]<br>**Performance:** [Accuracy: 77.68%, AUC: 83.1%]
13	Attentional network deficits in patients with migraine: behavioral and electrophysiological evidence	**Disorders:** [Migraine]<br>**Cohort:** [91 subjects]<br>**Data:** [EEG signal and behavioral data]<br>**Outcome:** [Migraine diagnosis]<br>**Validation:** [Leave-One-Out] [No train/test split]<br>**Core technical approach:** [Machine learning]

	**Performance:** [Accuracy: 77.9%, F1-score: 76.2%, Precision: 77.4%, Recall: 75.6%]
14 Advancing toward precision migraine treatment: Predicting responses to preventive medications with machine learning models based on patient and migraine features	**Disorders:** [Migraine]<br>**Cohort:** [4,260 subjects]<br>**Data:** [Headache clinical and medical records]<br>**Outcome:** [Migraine treatment response prediction]<br>**Validation:** [K-fold] [85/15 train/test split]<br>**Core technical approach:** [Deep and machine learning]<br>**Treatment period:** [3 months]<br>**Performance:** [*Beta-blockers:* AUC: 66.4%, Precision: 40%, Recall: 20%, Accuracy: 73%, F1-score: 27%. *Tricyclic antidepressants:* AUC: 61.1%, Precision: 43%, Recall: 60%, Accuracy: 60%, F1-score: 50%. *Topiramate:* AUC: 60.5%, Precision: 39%, Recall: 44%, Accuracy: 63%, F1-score: 41%. *Verapamil:* AUC: 67.3%, Precision: 38%, Recall: 77%, Accuracy: 60%, F1-score: 51%. *Gabapentin:* AUC: 62.8%, Precision: 39%, Recall: 32%, Accuracy: 66%, F1-score: 35%. *OnabotulinumtoxinA:* AUC: 58.1%, Precision: 36%, Recall: 28%, Accuracy: 63%, F1-score: 32%. *CGRP mAbs*: AUC: 82.5%, Precision: 65%, Recall: 57%, Accuracy: 80%, F1-score: 60%]
15 A Comparative Analysis of Stimuli Response among People with Migraine Classification: A Machine Learning Approach	**Disorders:** [Migraine]<br>**Cohort:** [39 subjects]<br>**Data:** [EEG signal data]<br>**Outcome:** [Migraine diagnosis]<br>**Validation:** [No validation set] [80/20 train/test split]<br>**Core technical approach:** [Machine learning]<br>**Performance:** [Accuracy: 99.74%]
16 Application Value of a Machine Learning Model in Predicting Mild Depression Associated with Migraine without Aura	**Disorders:** [Migraine]<br>**Cohort:** [178 subjects]<br>**Data:** [Headache clinical and medical records]<br>**Outcome:** [Migraine diagnosis]<br>**Validation:** [No validation set] [140/38 train/test split]<br>**Core technical approach:** [Statistical analysis and machine learning]<br>**Performance:** [AUC: 90.1%, Sensitivity: 85.7%, Specificity: 92.9%, Accuracy: 89.6%]
17 Deciphering Migraine Types: A Machine Learning Odyssey for Precision Prediction	**Disorders:** [Migraine]<br>**Cohort:** [400 subjects]<br>**Data:** [Headache clinical and medical records]<br>**Outcome:** [Migraine diagnosis]<br>**Validation:** [No validation set info] [80/20 train/test split]<br>**Core technical approach:** [Machine learning]<br>**Performance:** [Accuracy: 85%, Precision: 96.22%, Sensitivity: 55.5%, F1-score: 85%, MCC: 70.4%, Specificity: 96.22%, NPV: 96.22%, FNR: 44.4%]
18 Effect of Demographic Characteristics on Tension Type Headache and Migraine: A Machine Learning Based Analysis	**Disorders:** [Migraine and tension type headache]<br>**Cohort:** [173 subjects]<br>**Data:** [Headache clinical and medical records]<br>**Outcome:** [Migraine/ tension type headache diagnosis]<br>**Core technical approach:** [Machine learning]<br>**Validation:** [5-fold] [No train/test split]<br>**Performance:** [Accuracy: 73.99%, Precision: 73.4%, Recall: 73.4%, AUC: 73.3%]

19	Migraine aura discrimination using machine learning: an fMRI study during ictal and interictal periods	**Disorders:** [Migraine]<br>**Cohort:** [2 subjects]<br>**Data:** [fMRI image data]<br>**Outcome:** [Migraine stage diagnosis]<br>**Validation:** [10-fold] [No train/test split info]<br>**Core technical approach:** [Machine learning]<br>**Performance:** [Accuracy: 74.1-97.3%, AUC: 87-98%, Precision: 79-95%, Specificity: 75-86%, Recall: 83-96%, F1-score: 77-91%]
20	Altered brainstem-cortex activation and interaction in migraine patients: somatosensory evoked EEG responses with machine learning	**Disorders:** [Migraine]<br>**Cohort:** [342 subjects]<br>**Data:** [EEG signal data]<br>**Outcome:** [Migraine diagnosis]<br>**Validation:** [5-fold] [90/10 train/test split]<br>**Core technical approach:** [Machine learning]<br>**Performance:** [*Chronic migraine vs controls:* Accuracy: 76.2%, AUC: 89%. *Episodic migraine vs controls:* Accuracy: 87%, AUC: 88%. *Chronic vs episodic migraine:* Accuracy: 72.7%, AUC: 74%]
21	Frequency-Specific Alternations in the Amplitude of Fluctuations in Tension-Type Headache: A Machine Learning Study	**Disorders:** [Tension type headache]<br>**Cohort:** [64 subjects]<br>**Data:** [MRI and rs-fMRI image data]<br>**Outcome:** [Tension type headache diagnosis]<br>**Validation:** [10-fold] [No train/test split]<br>**Core technical approach:** [Machine learning]<br>**Performance:** [Accuracy: 93.81%, Sensitivity: 90.91%, Specificity: 96.67%, Precision: 96.77%, AUC: 97%]
22	Benchmarking State-of-the-Art Large Language Models for Migraine Patient Education: Performance Comparison of Responses to Common Queries	**Disorders:** [Migraine]<br>**Cohort:** [No human subjects were involved]<br>**Data:** [30 migraine related queries]<br>**Outcome:** [User experience on LLMs]<br>**Core technical approach:** [LLM assessment]<br>**Validation:** [5 LLMs recruited and 30 responses from each LLM assessed binary by 3 independent blinded physicians: 0: Inappropriate, 1: Appropriate]
23	Shifts in structural connectome organization in the limbic and sensory systems of patients with episodic migraine	**Disorders:** [Migraine]<br>**Cohort:** [88 subjects]<br>**Data:** [MRI image data]<br>**Outcome:** [Migraine diagnosis]<br>**Validation:** [5-fold] [No train/test split]<br>**Core technical approach:** [Machine learning]<br>**Performance:** [AUC: 90% ± 3%, Precision: 84% ± 5%, Recall: 84% ± 5%]
24	Preliminary External Validation Results of the Artificial Intelligence-Based Headache Diagnostic Model: A Multicenter Prospective Observational Study	**Disorders:** [Primary and secondary headache]<br>**Cohort:** [59 subjects]<br>**Data:** [Headache self-reported patient data]<br>**Outcome:** [Headache diagnosis]<br>**Validation:** [This is an already trained model which is described in the 8th row of the current table row. The model is just further evaluated with a new cohort as described in participants]<br>**Core technical approach:** [Machine learning]

		**Performance:** [Accuracy: 94.92%, Sensitivity: 98.21%, Specificity: 66.67%, Precision: 98.21%, F1-score: 98.21%]
25	An evolving machine-learning-based algorithm to early predict response to anti-CGRP monoclonal antibodies in patients with migraine	**Disorders:** [Migraine]<br>**Cohort:** [336 subjects]<br>**Data:** [Headache self-reported patient data]<br>**Outcome:** [Migraine treatment response prediction]<br>**Validation:** [80/20 train/validation set split] [External cohort of 93 patients used as test set]<br>**Core technical approach:** [Machine learning]<br>**Performance:** [Accuracy: 39.56-64.71%, AUC: 52.16-78.02%, Precision: 39.47-64.70%, Recall: 39.56-75.48%, F1-score: 40.58-60.62%]<br>**Treatment period:** [12 months]
26	Enhancing Migraine Diagnosis and Classification with TabNet: A Data-Driven Approach	**Disorders:** [Migraine]<br>**Cohort:** [400 subjects]<br>**Data:** [Headache clinical and medical records]<br>**Outcome:** [Migraine diagnosis]<br>**Validation:** [DL validation] [No train/test split info]<br>**Core technical approach:** [Deep learning]<br>**Performance:** [Accuracy: 98%, Precision: 99%, F1-score: 98%]
27	The Sophisticated Prognostication of Migraine Aura Using Machine Learning	**Disorders:** [Migraine]<br>**Cohort:** [400 subjects]<br>**Data:** [Headache clinical and medical records]<br>**Outcome:** [Migraine diagnosis]<br>**Validation:** [10-fold] [70/30 train/test split]<br>**Core technical approach:** [Machine learning]<br>**Performance:** [Accuracy: 99.5%]
28	Exploring potential neuroimaging biomarkers for the response to non-steroidal anti-inflammatory drugs in episodic migraine	**Disorders:** [Migraine]<br>**Cohort:** [118 participants]<br>**Data:** [rs-fMRI image data]<br>**Outcome:** [Migraine treatment response prediction]<br>**Validation:** [70/30 train/validation split] [External validation cohort used: 39 patients]<br>**Core technical approach:** [Machine learning]<br>**Performance:** [AUC: 63.1%, Balanced accuracy: 61.1%, Sensitivity: 80.8%, F1-score: 65.6%, PPV: 55.3%, NPV: 70.6%]
29	A hybrid fuzzy clustering approach for diagnosing primary headache disorder	**Disorder:** [Primary headache]<br>**Cohort:** [No information provided]<br>**Data:** [Headache self-reported patient data]<br>**Outcome:** [Headache diagnosis]<br>**Validation:** [2 test sets. Set 1: 579 subjects and Set 2: 132 subjects]<br>**Core technical approach:** [Fuzzy logic]<br>**Performance:** [Mean average accuracy: 84.3%]
30	MwoA auxiliary diagnosis via RSN-based 3D deep multiple instance learning with spatial attention mechanism	**Disorder:** [Migraine]<br>**Cohort:** [125 subjects]<br>**Data:** [rs-fMRI data]<br>**Outcome:** [Migraine diagnosis]<br>**Validation:** [DL validation] [No train/test split info provided]<br>**Core technical approach:** [Deep learning]

		**Performance:** [Accuracy: 88.8%, Precision: 91.3%, Recall: 88.3%, F1-score: 88.4%, AUC: 94.7%]
31	Diagnosing migraine from genome-wide genotype data: a machine learning analysis	**Disorder:** [Migraine]<br>**Cohort:** [43,197 subjects]<br>**Data:** [Genetic data]<br>**Outcome:** [Migraine diagnosis]<br>**Validation:** [10-fold] [90/10 train/test split]<br>**Core technical approach:** [Machine learning]<br>**Performance:** [AUC: 63%, Accuracy: 62%, Precision: 64%, Recall: 60%, F1-score: 57%,]

Table S5. New publications on data science included in the review. The table provides information regarding, where applicable, the headache disorder, the cohort, the data modality, the clinical outcome, the validation and testing dataset splitting, the technical methodology, the application used for gathering data, the trial period and the performance reported by the corresponding publication.

Nr	Title	Characteristics
1	Application of density estimation algorithms in analyzing co-morbidities of migraine	**Disorder:** [Migraine]<br>**Cohort:** [116,136 subjects]<br>**Data:** [Headache clinical and medical records]<br>**Outcome:** [Migraine analysis]<br>**Core technical approach:** [Statistical analysis]
2	Tracking and visualizing headache trends on a mobile or desktop website	**Disorder:** [Primary and secondary headache]<br>**Cohort:** [No information provided]<br>**Data:** [Headache self-reported patient data]<br>**Outcome:** [Disorder monitoring]<br>**Core technical approach:** [Data capture and visualization]<br>**Application:** [Website]
3	The recognition of migraine headache by designation of fuzzy expert system and usage of LFE learning algorithm	**Disorder:** [Migraine]<br>**Cohort:** [148 subjects]<br>**Data:** [Headache clinical and medical records]<br>**Outcome:** [Migraine diagnosis]<br>**Validation:** [80/20 train/test split]<br>**Core technical approach:** [Fuzzy logic]<br>**Performance:** [Accuracy: 97%, Precision: 80%, Sensitivity: 70%, Specificity: 94%]
4	The added value of an electronic monitoring and alerting system in the management of medication-overuse headache: A controlled multicenter study	**Disorder:** [MOH]<br>**Cohort:** [663 subjects]<br>**Data:** [Headache self-reported patient data]<br>**Outcome:** [Disorder monitoring]<br>**Core technical approach:** [Data capture and visualization]<br>**Trial period:** [6 months]<br>**Application:** [Comoestas Tool]
5	H-Diary: Mobile Application for Headache Diary and Remote Patient Monitoring	**Disorder:** [Primary and secondary headache]<br>**Cohort:** [No information provided]<br>**Data:** [Headache self-reported patient data]<br>**Outcome:** [Disorder monitoring]<br>**Core technical approach:** [Data capture and visualization]<br>**Application:** [H-Diary]<br>**Trial period:** [Not tried yet]
6	Understanding What People with Migraine Consider to be Important Features of Migraine Tracking: An Analysis of the Utilization of Smartphone-Based Migraine Tracking with a Free-Text Feature	**Disorder:** [Migraine]<br>**Cohort:** [288 subjects]<br>**Data:** [Headache self-reported patient data]<br>**Outcome:** [User experience]<br>**Core technical approach:** [Data capture and visualization]<br>**Trial period:** [90 days]<br>**Application: [**RELAXaHEAD**]**
7	Non-interacting, Non-opioid, and Non-barbiturate Containing Acute Medication Combinations in Headache: A Pilot Combinatorics	**Disorder:** [Primary and secondary headache]<br>**Cohort:** [No information provided]<br>**Data:** [DrugBank data]<br>**Outcome:** [Treatment analysis]<br>**Core technical approach:** [Statistical analysis]

	Approach Based on Drug-Bank Database	
8	Determining the Evolution of Headache Among Regular Users of a Daily Electronic Diary via a Smartphone App: Observational Study	**Disorder:** [Migraine]<br>**Cohort:** [1,545 subjects]<br>**Data:** [Headache self-reported patient data]<br>**Outcome:** [Disorder Monitoring]<br>**Core technical approach:** [Statistical analysis]<br>**Application:** [M-sense]<br>**Trial period:** [7 months]
9	Classifying migraine using PET compressive big data analytics of brain's μ-opioid and D2/D3 dopamine neurotransmission	**Disorder:** [Migraine]<br>**Cohort:** [61 subjects]<br>**Data:** [PET images]<br>**Outcome:** [Migraine diagnosis]<br>**Validation:** [10-fold] [80/20 training/testing set]<br>**Core technical approach:** [Statistical analysis]<br>**Performance:** [Mean replication accuracy: 93.77%, Mean replication sensitivity: 86.95%, Mean replication specificity: 99.78%]
10	Feasibility and Usability of a Mobile App-Based Interactive Care Plan for Migraine in a Community Neurology Practice: Development and Pilot Implementation Study	**Disorder:** [Migraine]<br>**Cohort:** [171 subjects]<br>**Data:** [Headache self-reported patient and physiological data]<br>**Outcome:** [User experience]<br>**Core technical approach:** [Statistical analysis]<br>**Application:** [MICP]<br>**Trial period:** [3 months]
11	Single Arm Feasibility Trial of a Mobile Application for Adolescent Migraine Management	**Disorder:** [Migraine]<br>**Cohort:** [18 cases]<br>**Data:** [HRV data]<br>**Outcome:** [User experience]<br>**Core technical approach:** [Statistical analysis]<br>**Application:** [EaseDay]<br>**Trial period:** [4 weeks]
12	Factors and Reasons Associated with Hesitating to Seek Care for Migraine: Results of the OVERCOME (US) Study	**Disorder:** [Migraine]<br>**Cohort:** [58,403 cases]<br>**Data:** [Self-reported patient data]<br>**Outcome:** [User experience]<br>**Core technical approach:** [Machine learning]<br>**Application:** [OVERCOME (US)]
13	Development and Evaluation of a Smartphone-Based Chatbot Coach to Facilitate a Balanced Lifestyle in Individuals with Headaches (BalanceUP App): Randomized Controlled Trial	**Disorder:** [Primary headache]<br>**Cohort:** [198 subjects]<br>**Data:** [Headache self-reported patient data]<br>**Outcome:** [User experience]<br>**Core technical approach:** [Statistical analysis]<br>**Application:** [BalanceUP]
14	Practical Experience with the Use of Electronic Headache Diaries and Video Consultations in Migraine Care from a Longitudinal Cohort Study	**Disorder:** [Migraine]<br>**Cohort:** [532 cases]<br>**Data:** [Headache self-reported patient data]<br>**Outcome:** [User experience]<br>**Core technical approach:** [Statistical analysis]<br>**Trial period:** [181 days on average]<br>**Application:** [Leiden Headache Center's e-diary]

15	Clinical decision support system using hierarchical fuzzy diagnosis model for migraine and tension-type headache based on International Classification of Headache Disorders, 3rd edition	**Disorder:** [Migraine and tension type headache]<br>**Cohort:** [705 subjects]<br>**Data:** [Headache clinical and medical records]<br>**Outcome:** [Migraine and tension type headache diagnosis]<br>**Validation:** [325 samples in retrospective set and 380 samples in prospective set]<br>**Core technical approach:** [Fuzzy logic]<br>**Evaluation:** [*Migraine:* Sensitivity: 97.71%, Specificity: 100.0%, PPV: 100.0%, NPV: 98.48%, Youden's J statistic: 0.9771. *Tension type headache:* Sensitivity: 98.57%, Specificity: 100.0%, PPV: 100.0%, NPV: 99.61%, Youden's J statistic: 0.9857]
16	Morphological similarity and white matter structural mapping of new daily persistent headache: a structural connectivity and tract-specific study	**Disorder:** [New daily persistent headache]<br>**Cohort:** [51 subjects]<br>**Data:** [MRI imaging data]<br>**Outcome:** [Headache analysis]<br>**Core technical approach:** [Statistical analysis]
17	Whole-brain functional gradients reveal cortical and subcortical alterations in patients with episodic migraine	**Disorders:** [Migraine]<br>**Cohort:** [100 subjects]<br>**Data:** [rs-fMRI image data]<br>**Outcome:** [Migraine analysis]<br>**Validation:** [5-fold] [No train/test split]<br>**Core technical approach:** [Machine learning]<br>**Performance:** [*Frequency:* ICC: 0.33 ± 0.13, MAE: 2.97±0.57. *Duration:* No significant prediction performance achieved]

Table S6. New publications on wearable devices included in the review. The table provides information regarding, where applicable, the headache disorder, the cohort, the data modality, the clinical outcome, the validation and testing dataset splitting, the technical methodology, wearable devices, the trial period and the performance reported by the corresponding publication.

Nr	Title	Characteristics
1	Using Sleep Time Data from Wearable Sensors for Early Detection of Migraine Attacks	**Disorder: [**Migraine]<br>**Cohort: [**7 subjects]<br>**Data:** [Physiological signals]<br>**Outcome:** [Forecasting of migraine attacks]<br>**Validation:** [5-fold] [80/20 train/test split]<br>**Core technical approach:** [Machine learning]<br>**Device:** [Empatica E4]<br>**Trial period:** [28.57, SD=3.57 days]<br>**Performance:** [Balanced accuracy: 84.1%, Sensitivity: 99.2%, Specificity: 71.5%]
2	Brain state monitoring for the future prediction of migraine attacks	**Disorder:** [Migraine]<br>**Cohort:** [24 subjects]<br>**Data:** [EEG signal data]<br>**Outcome:** [Migraine analysis]<br>**Core technical approach:** [Statistical analysis]<br>**Device:** [BrainStation by Neuroverse]<br>**Trial period:** [13.3, SD=1.9 days]
3	Biofeedback Treatment App for Pediatric Migraine: Development and Usability Study	**Disorder:** [Migraine]<br>**Cohort:** [10 subjects]<br>**Data:** [Physiological data]<br>**Outcome:** [User experience and treatment analysis]<br>**Core technical approach:** [Optimization]<br>**Trial period:** [2 weeks]<br>**Device:** [In-house developed sensor and app]
4	A Low Power Multi-Class Migraine Detection Processor Based on Somatosensory Evoked Potentials	**Disorder:** [Migraine]<br>**Cohort:** [No information provided]<br>**Data:** [SEP signals]<br>**Outcome:** [Forecasting of migraine attacks]<br>**Validation:** [57 subjects used for validation] [No train/test split info]<br>**Core technical approach:** [Machine learning]<br>**Trial period:** [No information provided]<br>**Device:** [CED 1401]<br>**Performance:** [Accuracy: 76.0%, Precision: 100%]
5	An 8.7 μJ/class. FFT accelerator and DNN-based configurable SoC for Multi-Class Chronic Neurological Disorder Detection	**Disorder:** [Migraine]<br>**Cohort:** [No information provided]<br>**Data:** [EEG signal data]<br>**Outcome:** [Forecasting of migraine attacks]<br>**Validation:** [DL validation] [No train/test split info]<br>**Core technical approach:** [Deep learning]<br>**Trial period:** [No information provided]<br>**Device:** [No information provided]<br>**Performance:** [Accuracy: 81.4%]
6	Feasibility of using "SMARTER" methodology for monitoring precipitating	**Disorder:** [Migraine]<br>**Cohort:** [30 subjects]<br>**Data:** [Headache self-reported patient and physiological data]

	conditions of pediatric migraine episodes	**Outcome:** [User experience and treatment analysis]<br>**Core technical approach:** [Data capture and visualization]<br>**Trial period:** [28 days]<br>**Device:** [Empatica Embrace and mEMA mobile app]
7	A Biofeedback App for Migraine: Development and Usability Study	**Disorder:** [Migraine]<br>**Cohort:** [18 subjects]<br>**Data:** [Headache self-reported patient and physiological data]<br>**Outcome:** [User experience and treatment analysis]<br>**Core technical approach:** [Data capture and visualization]<br>**Trial period:** [4 weeks]<br>**Device:** [Cerebri, Nordic Brain Tech AS]
8	Heartrate variability biofeedback for migraine using a smartphone application and sensor: A randomized controlled trial	**Disorder:** [Migraine]<br>**Cohort:** [52 subjects]<br>**Data:** [HRV signal data]<br>**Outcome:** [Treatment analysis and user experience]<br>**Core technical approach:** [Data capture and visualization]<br>**Trial period:** [8 weeks]<br>**Device:** [HeartMath]
9	A Prospective Real-World Study Exploring Associations Between Passively Collected Tracker Data and Headache Burden Among Individuals with Tension-Type Headache and Migraine	**Disorder:** [Migraine and tension type headache]<br>**Cohort:** [350 subjects]<br>**Data:** [Headache self-reported patient and physiological data]<br>**Outcome:** [TTH and Migraine analysis]<br>**Core technical approach:** [Statistical analysis]<br>**Trial period:** [12 weeks]<br>**Device:** [Fitbit and mobile]
10	Intelligent Digital Twins for Personalized Migraine Care	**Disorder:** [Migraine]<br>**Cohort:** [No information provided]<br>**Data:** [Multimodal]<br>**Outcome:** [Disorder monitoring]<br>**Core technical approach:** [Digital Twins]<br>**Trial period:** [No information provided]<br>**Device:** [No information provided]
11	Forecasting migraine with machine learning based on mobile phone diary and wearable data	**Disorder:** [Migraine]<br>**Cohort:** [18 subjects]<br>**Data: [**Headache self-reported patient and physiological data]<br>**Outcome:** [Forecasting of migraine attacks]<br>**Validation:** [75/25 training validation split, 3-fold] [75/25 training/testing split]<br>**Core technical approach:** [Machine learning]<br>**Trial period:** [295 days]<br>**Device:** [Cerebri, Nordic Brain Tech AS]<br>**Performance:** [Accuracy: 56.0%, AUC: 62.0%, Sensitivity: 0.0%, Specificity: 100.0%]
12	Machine Learning and Wearable Technology: Monitoring Changes in Biomedical Signal Patterns during Pre-Migraine Nights	**Disorders:** [Migraine]<br>**Cohort:** [10 subjects]<br>**Data:** [Physiological data]<br>**Outcome:** [Forecasting of migraine attacks]<br>**Validation:** [5-fold] [No train/test split]<br>**Core technical approach:** [Machine learning]<br>**Device:** [Empatica Embrace Plus]

		**Trial period:** [No information provided]<br>**Performance:** [Accuracy: 80.6%, Precision: 63.8%, Recall: 59.5%, F1-score: 60.7%]
13	sEMG Biofeedback for Episodic Migraines: A Pilot Randomized Clinical Trial	**Disorder:** [Migraine]<br>**Cohort:** [84 subjects]<br>**Data:** [Headache self-reported patient data]<br>**Outcome:** [Treatment analysis]<br>**Core technical approach:** [Statistical analysis]<br>**Device:** [JOGO Digital Therapeutics sEMG-BF]<br>**Trial period:** [6 weeks]
14	Patients with chronic cluster headache may show reduced activity energy expenditure on ambulatory wrist actigraphy recordings during daytime attacks	**Disorders:** [Cluster headache]<br>**Cohort:** [4 subjects]<br>**Data:** [Actigraphy and accelerometer data]<br>**Outcome:** [Cluster headache analysis]<br>**Core technical approach:** [Statistical analysis]<br>**Device:** [Empatica E4]<br>**Trial period:** [21 days]
